# Supplementary figures and images for: Achilles and tail tendons of perlecan exon 3 null heparan sulphate deficient mice display surprising improvement in tendon tensile properties and altered collagen fibril organisation compared to C57BL/6 wild type mice
Source: PeerJ. 2018 Jun 29;6:e5120. doi: 10.7717/peerj.5120 (PMC6056265; doi:10.7717/peerj.5120)

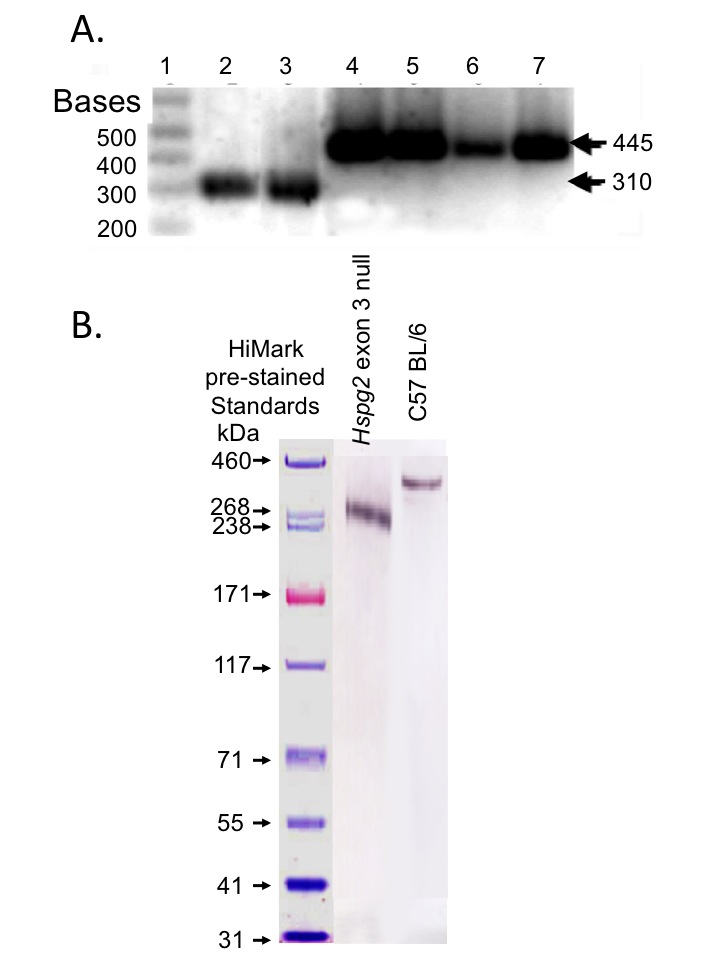

Supplement: Figure S1 — Specific regions of the mouse perlecan gene were amplified by PCR using genotyping primers recognising intron 2 of mouse Hspg2 (GTA GGG ACA CTT GTC ATC CT), exon 3 (CTG CCA AGG CCA TCT GCA AG) and Hspg2Δ3−∕Δ3− (AGG AGT AGA AGG TGG CGC GAA GG). The PCR amplified genomic DNA bands were separated and identified by 2% agarose gel electrophoresis (A). (1) Standard 1 kb DNA ladder, (2–3) WT genomic DNA, (4–7) Hspg2Δ3−∕Δ3− genomic DNA, with the expected 310 and 445 base pair products indicated. (B) Western blotting of 3–8% PAG gradient gel separated perlecan isolated from WT and Hspg2 exon 3 null skeletal muscle using perlecan C-terminal antibody H300 (Santa Cruz) and alkaline phosphatase conjugated secondary antibody, NBT/BCIP for colour detection. [file peerj-06-5120-s002.jpg]
